# Role of injection laryngoplasty in preventing post-stroke aspiration pneumonia, case series report

**DOI:** 10.1097/MD.0000000000019220

**Published:** 2020-02-14

**Authors:** Yeon Jae Han, Yong Jun Jang, Geun-Young Park, Young Hoon Joo, Sun Im

**Affiliations:** a Department of Rehabilitation Medicine, Bucheon St. Mary's Hospital, College of Medicine; b Department of Rehabilitation Medicine, Graduate School; c Department of Otolaryngology-Head and Neck Surgery, College of Medicine, The Catholic University of Korea, Seoul, Republic of Korea.

**Keywords:** aspiration, case report, cough, laryngoplasty, stroke, vocal fold dysfunction

## Abstract

**Introduction:**

Injection laryngoplasty is a common procedure for patients with vocal fold dysfunction, but the literature on its benefits has been mainly focused on those related to structural lesions or laryngeal nerve involvement. Stroke patients may be at increased risk of aspiration due to insufficient vocal fold motion. However, how injection laryngoplasty can be of benefit in stroke patients has not been reported yet.

**Patient concerns:**

Six chronic stroke patients with long-standing swallowing difficulties and who showed severe aspiration despite long-term swallowing rehabilitation.

**Diagnosis:**

Laryngoscope evaluation revealed insufficient glottic closure as the cause of aspiration.

**Interventions:**

Injection laryngoplasty was done per-orally under local anaesthesia with calcium hydroxylapatite (Radiesse Voice, 1–1.5 mL) in an office setting. Respiratory pressures and peak cough flows were assessed at baseline and at 2 weeks follow-up.

**Outcomes:**

At 2 weeks, the mean peak cough flow (Δ = +95.09 L/min) increased significantly after the procedure. The maximal expiratory (Δ = +18.40 cm H_2_O) and inspiratory (Δ = +20.20 cm H_2_O) pressures also improved, indicating that injection laryngoplasty was effective in augmenting respiratory and cough parameters. All cases showed improvement in the Functional Oral Intake Scale (Δ = +4). Feeding tubes were successfully removed.

**Conclusion:**

Injection laryngoplasty proved to be both successful and safe in improving glottic closure with immediate results in those who had failed to show a positive response after long-term swallowing rehabilitation. The positive and dramatic clinical outcomes were observed through changes in the coughing force. Our case series support the use of injection larygnoplasty as a powerful adjunctive treatment method to prevent aspiration pneumonia in post-stroke patients with vocal fold insufficiency. Pre- and post-injection peak cough flow changes may reflect improvement in glottic closure and indicate the safety of swallowing with reduced risk of aspiration.

## Abstract 2

Supplemental Digital Content is available in the text

## 1 Introduction

The etiology of vocal fold dysfunction may include direct structural involvement of the recurrent laryngeal nerve, either due to injury or direct compression from a local or metastatic disease. In rare cases, neurologic diseases such as Parkinson's disease, motor neuron disease, and stroke; especially those involving the brainstem medullary area; may also cause vocal fold dysfunction.^[1,2]^

Proper glottal closure protects the airway from aspiration of respiratory secretions or food materials. Also, it provides an adequately high expiratory flow to remove aspirated material from the airway.^[3–5]^ Therefore, irrespective of the etiology, glottal incompetence may predispose patients to weak tussive reflex, thus leading to weakened cough and increased risk of aspiration. In stroke patients, who are vulnerable to aspiration pneumonia, glottic insufficiency may lead to catastrophic respiratory consequences.^[6,7]^

The optimal management for vocal fold dysfunction remains controversial. Many different variables should be considered, including the level of injury severity, a potential for nerve recovery and patients’ comorbidities.^[8]^ Surgical correction is sometimes indicated in patients with moderate to severe dysphagia or with multiple hospitalizations from aspiration pneumonia.^[5]^ However, post-stroke patients are at increased risk to undergo general anesthesia and receive surgical correction. Injection laryngoplasty, by contrast, is a safe procedure that can be performed in an office-based setting. Past studies have shown this procedure to be less invasive but at the same time effective in restoring glottis competence in vocal fold dysfunction related to postsurgical injury or head and neck malignancies.^[1,8–10]^ However, the role of injection laryngoplasty in stroke patients has not been reported.

The following cases demonstrate how the injection procedure can be an effective and feasible treatment in those with post-stroke vocal fold dysfunction with dramatic effects within 2 weeks.

## 2 Case report

We report six cases of post-stroke dysphagia patients who had undergone office-based injection laryngoplasty from February 2015 to December 2017. All patients had dysphagia related to first ischemic or hemorrhagic stroke. Three of them had been diagnosed with brain hemorrhage; two with a previous history of intubation, and the other three had been diagnosed with cerebral infarction. The mean ± standard deviation duration of dysphagia was 10.5 ± 8.38 months with a variable range from 3 to 23 months. The demographic and clinical characteristics of the patients are shown in Table 1. All six patients has provided informed consent for publication of the case (Supplemental Digital Contents 1–6). The institutional review board approved of this study.

**Table 1:**
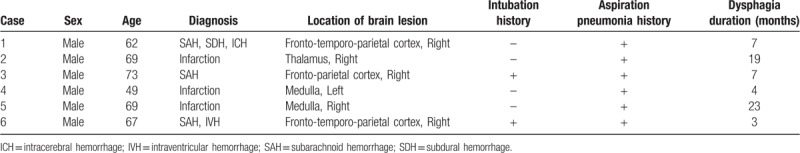
Participant description.

All patients were confirmed to have vocal fold dysfunction through laryngoscopy and stroboscopy by the laryngologist and/or Fiberoptic endoscopic evaluation of swallowing by a qualified specialist. All patients had no previous concomitant vocal fold lesions or underlying disease other than stroke, that could cause vocal fold paralysis. Other co-interventions were not performed during the treatment period. All patients had already received conventional dysphagia rehabilitation program, at least 30 sessions in total but had persistent aspiration. All patients had a previous record of recurrent aspiration pneumonia that required antibiotic treatment. Dysphagia therapy sessions remained unchanged after the procedures.

Injection laryngoplasty was performed by the surgeon at the Department of Otolaryngology-Head and Neck Surgery with 12 years of experience. Injection laryngoplasty was done per-orally under local anesthesia with calcium hydroxylapatite (Radiesse Voice, 1–1.5 mL; Merz Aesthetics, Inc., Franksville, WI) in an office setting. All patients had undergone the injection laryngoplasty successfully without any complications.

The following parameters that included peak cough flow (PCF), maximal inspiratory pressure (MIP), maximal expiratory pressure (MEP) were collected at two time points; at baseline and 2-weeks after the procedure. Penetration-Aspiration Scale (PAS),^[11]^ Functional Oral Intake Scale (FOIS),^[12]^ and Modified Barium Swallow Impairment Profile^TM^© (MBSImP ^TM©^)^[13]^ were collected at three time points; at baseline, 2-weeks and 3-months after the procedure but the follow-up schedules varied on participant's availability.

The PCF had been collected after individuals were asked to perform a quick, short and powerful cough on the peak flow meter (Micropeak; Carefusion, Corp., San Diego, CA), in compliance with the American Thoracic Society (ATS) standards. All patients had completed three trials of maximal voluntary cough. According to the ATS spirometry standard, the two highest recordings were evaluated. The MIP and MEP, known for correlation with cough capacity,^[14]^ was also assessed following ATS guideline, each subject performed a forceful inspiration after expiration or expiration after full inspiration, with their nose closed by a nose-clip.^[15]^ Participants were carefully instructed on the protocol, and the physical therapists simulated the procedures before the measurements were made. Videofluoroscopic swallowing study (VFSS) was performed in accordance to Logemann protocol by a trained specialist in interpreting the findings.^[16]^ From the VFSS findings, the level and severity of aspiration were scored by the PAS, the level of oral intake by the FOIS and the swallowing parameters by the MBSImP^TM©^. The total and the oral and pharyngeal impairment stages subscores were analyzed.

Pre- and post-outcomes were compared for descriptive analyses. Wilcoxon signed rank test was performed with 95% confidence intervals. One-sided *P* = .05 was used to indicate statistical significance. All statistical analyses were performed using R and RStudio software, version 1.1.423 (R) (RStudio, Inc., Boston, MA).

**Figure 1:**
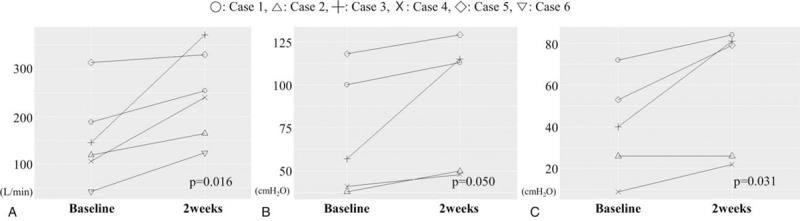
Measurements of the (A) PCF (B) MEP and (C) MIP at baseline and post-2-weeks after injection laryngoplasty obtained from the six participants. Though PCF data were available from all cases, one case had missing data for post-intervention MEP and MIP. All cases showed significant improvement in the parameters compared to baseline at post-2-weeks. MEP = maximal expiratory pressure, MIP = maximal inspiratory pressure, PCF = peak cough flow.

Results from the six patients showed the mean PCF to increase significantly after the procedure (baseline = 152.58 ± 92.53 L/min; 2-weeks, Δ = +95.09 L/min; *P*-value = .016). The mean MEP showed significant improvement (baseline = 70.80 ± 36.18 cm H_2_O; 2-weeks, Δ = +18.40 cm H_2_O; *P*-value = .050) and mean MIP also improved (baseline = 40.00 ± 24.24 cm H_2_O; 2-weeks, Δ = +20.20 cm H_2_O; *P*-value = .031), indicating that injection laryngoplasty was effective in augmenting respiratory and cough parameters (Fig. 1). Though PCF data were all available for all six patients, one patient had missing data for post-intervention MIP and MEP.

**Figure 2:**
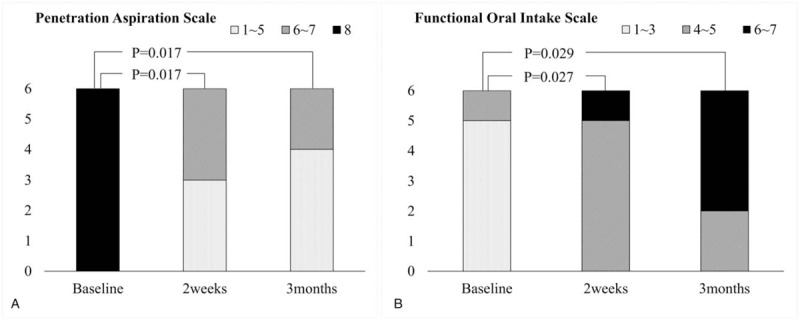
Measurements of the (A) PAS and (B) FOIS at baseline, post-2-weeks and post-3-months visits. PAS and FOIS showed significant improvement compared to baseline. FOIS = functional oral intake scale, PAS = penetration-aspiration scale.

Swallowing performance also showed significant changes. At baseline, median (range) PAS was 8 (8–8) and reduced to PAS of 6 (2–7) after 2-weeks (*P*-value = .017), with two patients even showing immediate improvements up to PAS 2 and 3. At post-3-months, median PAS was reduced to 1.5 (1–7) (*P*-value = .017), and four patients showed improvements up to PAS 1 and 2 (Fig. 2A).

In contrast to baseline FOIS levels in which the majority of patients were on tube feedings (FOIS < 4) with a median (range) score of FOIS 1 (1–5), feeding tubes were successfully removed with significant changes of the FOIS score (Δ = +4, *P*-value = .027) after 2-weeks. Additionally, at three-month follow-up, four of six patients no longer needed modifying food or fluid with FOIS ≥ 6 (Fig. 2B).

**Figure 3:**
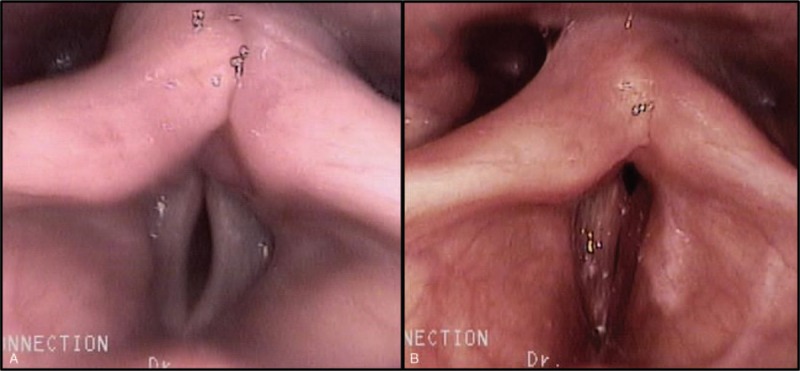
Laryngoscope images showing a noticeable glottic gap before the laryngoplasty procedure (A), and improved glottic closure after the procedure (B).

The total MBSImP^TM©^ scores (baseline = 26.5 [17–34]) improved significantly 2-weeks after the procedure, (Δ = −4.5, *P*-value = .017), especially in the total pharyngeal impairment scores (Δ = −2.5, *P*-value = .017), in the category for “Laryngeal Vestibular Closure” (Δ = −1.5, *P*-value = .016) and “Pharyngeal Residue” (Δ = −1, *P*-value = .044). At 3-months follow up, significant improvements were observed in the total MBSImP^TM©^ scores (Δ = −19.5, *P*-value = .001), especially in the pharyngeal total scores (Δ = −6, *P*-value = .039), in the “Laryngeal Vestibular Closure” (Δ = −1, *P*-value = .012) and “Pharyngeal Residue” (Δ = −1, *P*-value = .050), “Laryngeal Elevation” (Δ = −0.5, *P*-value = .027) and “Anterior Hyoid Excursion” (Δ = −1, *P*-value = .012) categories. Follow-up laryngoscope findings showed improved glottic closure (Fig. 3).

A detailed medical record review after 1 year of the procedures showed that no long-term discomfort after the injections were present. All patients no longer showed aspiration signs and recovered to FOIS levels of 7 with no further events of aspiration pneumonia, except one patient who had recurrent cerebral infarction during the follow-up period.

## 3 Discussion

In this case-series of six stroke patients with chronic swallowing disturbance and aspiration, we have shown that injection laryngoplasty leads to improvement of glottic closure, thus resulting in reduced aspiration and increased cough force. The procedure proved to be safe with no complications. Also, no further episodes of aspiration pneumonia were observed after the injections. This case-series demonstrates that injection laryngoplasty could be considered as a powerful adjunctive treatment with fast results to improve coughing and swallowing function in post-stroke patients with glottis insufficiency.

The importance of proper glottic closure during swallowing is crucial. Glottis insufficiency causes a constant pressure leak into the airway that can make swallowing more difficult. Also, proper glottic closure is crucial to generate a cough. Therefore, glottis insufficiency diminishes cough strength, thereby impeding one of the central mechanisms of airway cleansing and protection.^[4]^ Transcutaneous injection laryngoplasty improve glottal incompetence by repositioning of a patient's vocal folds toward the midline, allowing contact between the affected side and the normal side.^[17]^ The procedure allows proper contact of vocal folds and full glottic closure to produce proper voice, to swallow safe and to generate a strong cough.

Past reports had highlighted the efficacy of this procedure in patients with structural lesions, such as those related to idiopathic iatrogenic, or related to malignancies.^[1,8–10]^ But the efficacy in those with post-stroke dysphagia and its beneficial effects in augmenting the cough force are lacking.^[1,18]^ A strong cough offers protection from aspiration-related pneumonia, and there is evidence available to support its crucial role in the acute stroke, but augmenting the cough force can be challenging.^[19]^ To the best of our knowledge, our case-series report is the first to support the notion that injection laryngoplasty, may be considered as a useful adjunctive treatment method to augment the cough and respiratory strength, thereby leading to reduced aspiration and improved swallowing in stroke patients. In consideration that prevention of aspiration is a crucial issue in stroke patients, a more wide-spread application of this technique to stroke patients seems promising.

The reported technical success is 97% with low rates of complication. ^[17]^ In accordance, no complications were observed after the injection. The procedures were well tolerated with no adverse events. The ideal timing to perform injection larygnoplasty is still unclear, although some support early injection.^[20]^ In our study, the time from the onset of stroke to the procedure varied from 3 to 23 months. Four of the cases were in their chronic state despite having already received conventional therapies before the injection laryngoplasty. Therefore, the dramatic improvement seen after the injections in these chronic post-stroke dysphagia patients is noteworthy.

This study had several limitations, including the small sample size, absence of a control group of patients who did not undergo the procedure and the short follow-up period. Also, the patient's self-perception of improvement after the procedure and change of voice quality using the GRBAS (grade, roughness, breathiness, asthenia, and strain) were not included.^[21]^ Despite potential reporting biases and small patient numbers, this study has shown the results after the procedure in stroke patients to be consistent and supported by instrumental assessment with beneficial outcomes. Future prospective trials with a large sample size with subgroup analysis on the ideal timing of this procedure are warranted.

## 4 Conclusion

According to our six cases with post-stroke vocal fold dysfunction, injection laryngoplasty proved to be an effective, feasible treatment that led to improved glottic closure, subsequently resulting in higher PCF and improved airway closure. The outcome all led to reduced aspiration and improved swallowing function. The results of this report support the use of injection laryngoplasty in stroke patients who manifest with a high risk of aspiration due to insufficient glottic closure.

## Author contributions

**Conceptualization:** Sun Im.

**Formal analysis:** Yong Jun Jang.

**Supervision:** Geun-Young Park, Young Hoon Joo.

**Writing – original draft:** Yeon Jae Han.

Sun Im orcid: 0000-0001-8400-4911.

## Supplementary Material

Supplemental Digital Content
