# Supplementary material for: Role of injection laryngoplasty in preventing post-stroke aspiration pneumonia, case series report
Source: Medicine (Baltimore). 2020 Feb 14;99(7):e19220. doi: 10.1097/MD.0000000000019220 (PMC7035062; doi:10.1097/MD.0000000000019220)
Supplement: Supplemental Digital Content [file medi-99-e19220-s001.pdf]

Medicine®

PATIENT PHOTOGRAPHIC AUTHORIZATION, RELEASE AND DISCHARGE

I, Han Bum-Hum, give my consent and authorize the photograph(s) and/or video featuring my likeness to be published in *Medicine®*, a Wolters Kluwer publication. I understand that such imaging records may be published by *Medicine®* and/or any party acting under the license and authority of *Medicine®* in any print, visual, electronic or broadcast media, specifically including, but not limited to, medical journals and textbooks, scientific presentations and teaching courses and Internet websites, for the purpose of informing the medical profession or the general public about plastic surgery methods, results, issues, trends, concerns and similar matters. I further understand that the imaging records shall become the property of *Medicine®*.

I understand that I may refuse to sign this authorization and such refusal will have no effect on the medical treatment I receive.

I release and discharge *Medicine®*, and all parties acting under their license and authority from all rights that I may have in the imaging records and from any claim that I may have relating to such use in publication, including any claim for payment in connection with distribution or publication of the imaging records in any medium or any claim arising from the distribution or publication by any third party.

I hereby warrant that I am over twenty-one years of age, and competent to contract in my own name.

I grant this consent as a voluntary contribution in the interest of public education and certify that I have read the above Authorization, Release and Discharge and fully understand its terms.

Patient 한범흠 Date 2018. 11. 08

WITNESS/PHYSICIAN: SUN ZM

I have read the above Authorization, Release, and Discharge. I am the parent, guardian or conservator of Han Bum-Hum, a minor. I am authorized to sign this consent on his/her behalf and I grant this consent as a voluntary contribution in the interest of public education.

Parent/Guardian ______________________ Date ______________________

Medicine®

PATIENT PHOTOGRAPHIC AUTHORIZATION, RELEASE AND DISCHARGE

I, Lee Hang-Sub, give my consent and authorize the photograph(s) and/or video featuring my likeness to be published in *Medicine*®, a Wolters Kluwer publication. I understand that such imaging records may be published by *Medicine*® and/or any party acting under the license and authority of *Medicine*® in any print, visual, electronic or broadcast media, specifically including, but not limited to, medical journals and textbooks, scientific presentations and teaching courses and Internet websites, for the purpose of informing the medical profession or the general public about plastic surgery methods, results, issues, trends, concerns and similar matters. I further understand that the imaging records shall become the property of *Medicine*®.

I understand that I may refuse to sign this authorization and such refusal will have no effect on the medical treatment I receive.

I release and discharge *Medicine*®, and all parties acting under their license and authority from all rights that I may have in the imaging records and from any claim that I may have relating to such use in publication, including any claim for payment in connection with distribution or publication of the imaging records in any medium or any claim arising from the distribution or publication by any third party.

I hereby warrant that I am over twenty-one years of age, and competent to contract in my own name.

I grant this consent as a voluntary contribution in the interest of public education and certify that I have read the above Authorization, Release and Discharge and fully understand its terms.

Patient 이지섭 / 이항섭 Date 2018. 11. 05

WITNESS/PHYSICIAN: SUN ZM

I have read the above Authorization, Release, and Discharge. I am the parent, guardian or conservator of Lee Hang-Sub, a minor. I am authorized to sign this consent on his/her behalf and I grant this consent as a voluntary contribution in the interest of public education.

Parent/Guardian 이지섭 Date 2018. 11.05

Medicine®

PATIENT PHOTOGRAPHIC AUTHORIZATION, RELEASE AND DISCHARGE

I, Jeong Byeong-Ju, give my consent and authorize the photograph(s) and/or video featuring my likeness to be published in *Medicine®*, a Wolters Kluwer publication. I understand that such imaging records may be published by *Medicine®* and/or any party acting under the license and authority of *Medicine®* in any print, visual, electronic or broadcast media, specifically including, but not limited to, medical journals and textbooks, scientific presentations and teaching courses and Internet websites, for the purpose of informing the medical profession or the general public about plastic surgery methods, results, issues, trends, concerns and similar matters. I further understand that the imaging records shall become the property of *Medicine®*.

I understand that I may refuse to sign this authorization and such refusal will have no effect on the medical treatment I receive.

I release and discharge *Medicine®*, and all parties acting under their license and authority from all rights that I may have in the imaging records and from any claim that I may have relating to such use in publication, including any claim for payment in connection with distribution or publication of the imaging records in any medium or any claim arising from the distribution or publication by any third party.

I hereby warrant that I am over twenty-one years of age, and competent to contract in my own name.

I grant this consent as a voluntary contribution in the interest of public education and certify that I have read the above Authorization, Release and Discharge and fully understand its terms.

Patient [illegible] / [illegible] Date 2018.11.03

WITNESS/PHYSICIAN: SUN ZM [illegible]

I have read the above Authorization, Release, and Discharge. I am the parent, guardian or conservator of Jeong Byeong-Ju, a minor. I am authorized to sign this consent on his/her behalf and I grant this consent as a voluntary contribution in the interest of public education.

Parent/Guardian [illegible] Date 2018.11.03

Medicine®

PATIENT PHOTOGRAPHIC AUTHORIZATION, RELEASE AND DISCHARGE

I, NYAM SUREN, give my consent and authorize the photograph(s) and/or video featuring my likeness to be published in *Medicine®*, a Wolters Kluwer publication. I understand that such imaging records may be published by *Medicine®* and/or any party acting under the license and authority of *Medicine®* in any print, visual, electronic or broadcast media, specifically including, but not limited to, medical journals and textbooks, scientific presentations and teaching courses and Internet websites, for the purpose of informing the medical profession or the general public about plastic surgery methods, results, issues, trends, concerns and similar matters. I further understand that the imaging records shall become the property of *Medicine®*.

I understand that I may refuse to sign this authorization and such refusal will have no effect on the medical treatment I receive.

I release and discharge *Medicine®*, and all parties acting under their license and authority from all rights that I may have in the imaging records and from any claim that I may have relating to such use in publication, including any claim for payment in connection with distribution or publication of the imaging records in any medium or any claim arising from the distribution or publication by any third party.

I hereby warrant that I am over twenty-one years of age, and competent to contract in my own name.

I grant this consent as a voluntary contribution in the interest of public education and certify that I have read the above Authorization, Release and Discharge and fully understand its terms.

Patient MYGMAR SUREN / NYAMSUREN Date 2018.10.31

WITNESS/PHYSICIAN: SUN ZM [signature]

I have read the above Authorization, Release, and Discharge. I am the parent, guardian or conservator of NYAM SUREN, a minor. I am authorized to sign this consent on his/her behalf and I grant this consent as a voluntary contribution in the interest of public education.

Parent/Guardian MYGMARSUREN Date 2018.10.31

Medicine®

PATIENT PHOTOGRAPHIC AUTHORIZATION, RELEASE AND DISCHARGE

I, Kim Jeong-Sul, give my consent and authorize the photograph(s) and/or video featuring my likeness to be published in *Medicine®*, a Wolters Kluwer publication. I understand that such imaging records may be published by *Medicine®* and/or any party acting under the license and authority of *Medicine®* in any print, visual, electronic or broadcast media, specifically including, but not limited to, medical journals and textbooks, scientific presentations and teaching courses and Internet websites, for the purpose of informing the medical profession or the general public about plastic surgery methods, results, issues, trends, concerns and similar matters. I further understand that the imaging records shall become the property of *Medicine®*.

I understand that I may refuse to sign this authorization and such refusal will have no effect on the medical treatment I receive.

I release and discharge *Medicine®*, and all parties acting under their license and authority from all rights that I may have in the imaging records and from any claim that I may have relating to such use in publication, including any claim for payment in connection with distribution or publication of the imaging records in any medium or any claim arising from the distribution or publication by any third party.

I hereby warrant that I am over twenty-one years of age, and competent to contract in my own name.

I grant this consent as a voluntary contribution in the interest of public education and certify that I have read the above Authorization, Release and Discharge and fully understand its terms.

Patient 양점순 / 김정술 Date 2018. 11. 02

WITNESS/PHYSICIAN: SUN 2M [signature]

I have read the above Authorization, Release, and Discharge. I am the parent, guardian or conservator of Kim Jeong-Sul, a minor. I am authorized to sign this consent on his/her behalf and I grant this consent as a voluntary contribution in the interest of public education.

Parent/Guardian 양점순 Date 2018. 11. 02

Medicine®

PATIENT PHOTOGRAPHIC AUTHORIZATION, RELEASE AND DISCHARGE

I, Shin Eung-cheol, give my consent and authorize the photograph(s) and/or video featuring my likeness to be published in *Medicine®*, a Wolters Kluwer publication. I understand that such imaging records may be published by *Medicine®* and/or any party acting under the license and authority of *Medicine®* in any print, visual, electronic or broadcast media, specifically including, but not limited to, medical journals and textbooks, scientific presentations and teaching courses and Internet websites, for the purpose of informing the medical profession or the general public about plastic surgery methods, results, issues, trends, concerns and similar matters. I further understand that the imaging records shall become the property of *Medicine®*.

I understand that I may refuse to sign this authorization and such refusal will have no effect on the medical treatment I receive.

I release and discharge *Medicine®*, and all parties acting under their license and authority from all rights that I may have in the imaging records and from any claim that I may have relating to such use in publication, including any claim for payment in connection with distribution or publication of the imaging records in any medium or any claim arising from the distribution or publication by any third party.

I hereby warrant that I am over twenty-one years of age, and competent to contract in my own name.

I grant this consent as a voluntary contribution in the interest of public education and certify that I have read the above Authorization, Release and Discharge and fully understand its terms.

Patient 신응철 Date 2018.11.05

WITNESS/PHYSICIAN: SUN ZM [signature]

I have read the above Authorization, Release, and Discharge. I am the parent, guardian or conservator of Shin Eung-Cheol, a minor. I am authorized to sign this consent on his/her behalf and I grant this consent as a voluntary contribution in the interest of public education.

Parent/Guardian ____________________ Date ____________________
